# Unraveling CCL20's role by regulating Th17 cell chemotaxis in experimental autoimmune prostatitis

**DOI:** 10.1111/jcmm.18445

**Published:** 2024-05-27

**Authors:** Cheng Zhang, Shun Xu, Rui‐Jie Hu, Xian‐Hong Liu, Shao‐Yu Yue, Xiao‐Ling Li, Bang‐Shun Dai, Chao‐Zhao Liang, Chang‐Sheng Zhan

**Affiliations:** ^1^ Department of Urology The First Affiliated Hospital of Anhui Medical University Hefei China; ^2^ Institute of Urology Anhui Medical University Hefei China; ^3^ Anhui Province Key Laboratory of Genitourinary Diseases Anhui Medical University Hefei China

**Keywords:** CCL20/CCR6 axis, experimental autoimmune prostatitis, IL‐17A, MAPK, NF‐κB, PI3K, Th17

## Abstract

Chronic prostatitis and chronic pelvic pain syndrome (CP/CPPS), a prevalent urological ailment, exerts a profound influence upon the well‐being of the males. Autoimmunity driven by Th17 cells has been postulated as a potential factor in CP/CPPS pathogenesis. Nonetheless, elucidating the precise mechanisms governing Th17 cell recruitment to the prostate, triggering inflammation, remained an urgent inquiry. This study illuminated that CCL20 played a pivotal role in attracting Th17 cells to the prostate, thereby contributing to prostatitis development. Furthermore, it identified prostate stromal cells and immune cells as likely sources of CCL20. Additionally, this research unveiled that IL‐17A, released by Th17 cells, could stimulate macrophages to produce CCL20 through the NF‐κB/MAPK/PI3K pathway. The interplay between IL‐17A and CCL20 establishes a positive feedback loop, which might serve as a critical mechanism underpinning the development of chronic prostatitis, thus adding complexity to its treatment challenges.

## 1 INTRODUCTION

Prostatitis is a common urological condition. According to the NIH classification, it could be categorized into four types,^1^ with Type III prostatitis, known as chronic prostatitis and chronic pelvic pain syndrome (CP/CPPS), standing as the predominant form, constituting a minimum of 90% of prostatitis diagnoses. In China, approximately 8.4% of males were afflicted by CP/CPPS.^2^ Shockingly, a survey reported a CP/CPPS prevalence as high as 25.3% among Chinese men aged 40–81, with limited clinical interventions showing limited effectiveness.^3^ Because of the unclear pathology of CP/CPPS, the treatment of CP/CPPS remained challenging. Scholars have put several hypotheses in an attempt to reveal its pathogenesis, and the prevailing viewpoint suggested that CP/CPPS may be associated with oxidative stress, autoimmune mechanisms and other factors.^4^

Within the realm of autoimmune maladies, Th17 cells have been recognized for their pivotal role due to their capacity to generate the inflammatory cytokine IL‐17A.^5^, ^6^ The cell membrane of Th17 consistently exhibit high levels of the chemokine receptor CCR6, thereby endowing CCR6 with a pivotal role in their recruitment to sites of inflammation.^7^, ^8^, ^9^ CCL20, alternatively known as macrophage inflammatory protein‐3α (MIP‐3α), liver and activation‐regulated chemokine (LARC) or Exodus‐1, is the unique ligand for CCR6.^10^, ^11^, ^12^ The promoter region of CCL20 harbours a nuclear factor (NF)‐κB binding site, emphasizing the crucial role of NF‐κB in inducing the expression of CCL20.^12^, ^13^ Additionally, IL‐17A has also been confirmed to possibly promote the expression of CCL20 through the MAPK or PI3K signalling pathway.^14^ Previous research has demonstrated that the inflammation site of autoimmunity disease existed specific cells that could secret CCL20.^15^, ^16^ Bae et al. have proposed that IL‐17A may potentially stimulate these cells to generate an increased quantity of CCL20.^15^, ^16^, ^17^ The binding of CCL20 and CCR6 activates a chemotactic response, recruiting CCR6^+^ cells, particularly Th17, to the site of inflammation.^10^, ^11^, ^18^, ^19^, ^20^ The recruitment of Th17 cells via the CCL20/CCR6 axis was associated with development of autoimmune diseases, thereby rendering the CCL20/CCR6 axis a prospective novel target for the management of autoimmune conditions, inclusive of CP/CPPS.

Here, we have delved into the possibility of the CCL20/CCR6 axis in recruiting Th17 cells to the prostate, consequently leading to experimental autoimmune prostatitis (EAP). Additionally, we investigate the potential sources and production mechanisms of CCL20. Our findings suggested that the interaction between the CCL20/CCR6 axis and IL‐17A initiates a vicious cycle, underscoring the potential of the CCL20/CCR6 axis as a promising therapeutic target for CP/CPPS.

## 2 MATERIALS AND METHODS

### 2.1 Mice and EAP induction

Six to eight‐week‐old male non‐obese diabetic (NOD)‐shiltjnju mice were obtained from the Nanjing Biomedical Research Institute of Nanjing University (Nanjing, China) and domiciled in the animal house with specific pathogen‐free (SPF) grade standards. All animal protocols were approved by the Committee for Animal Care and Use of the Animal Center of Anhui Medical University (No. LLSC20200282). The EAP mice were effectively established with the previous papers.^21^, ^22^ The prostate antigens (PAgs) were extracted from the prostate of Sprague–Dawley (SD) rats. Equal volumes of PAgs or a saline solution were meticulously emulsified within complete Freund's adjuvant (CFA; Sigma‐Aldrich, F5881) until mixtures exhibited immobility in aqueous surroundings. Mice were subcutaneously immunized at various sites, including all footpads and the base of the tails, using PAgs (300 mg/mouse, EAP group) or phosphate buffer solution (PBS; control group) emulsified within CFA, with a combined volume of 200 μL per mouse. Following the experimental protocol, mice underwent immunization on days 0 and 28, followed by euthanasia on day 42. A minimum of four NOD mice were employed in each group.

Anti‐mouse CCL20 neutralizing antibodies (20 μg/mouse; R&D system, AF760) or anti‐mouse IL‐17A neutralizing antibodies (10 μg/mouse; R&D system, MAB421) were individually administered intraperitoneally to separate groups of mice. This administration occurred one day before the induction of EAP and subsequently on days 6, 13, 20, 27, 34 and 41 post‐induction. A portion of the EAP mice received intraperitoneal injections of recombinant mouse CCL20 (rCCL20, 200 μg/mouse; Peprotech, 250–27) or recombinant mouse IL‐17A (100 μg/mouse; Peprotech, 210–17) on a daily basis for 1 week following both the initial and secondary immunizations. These recombinant cytokines and antibodies were reconstituted or diluted with aseptic saline.

### 2.2 Mechanical allodynia

As previously described, the abdominal allodynia of mice in different groups was evaluated using von Frey force filaments.^22^, ^23^, ^24^ On the 40th day post‐immunization, they were introduced into a novel environment, consisting of individual plastic chambers featuring a wire grid floor (6 cm × 10 cm × 12 cm), allowing them to acclimate for a period exceeding 30 min. Subsequent to the aforementioned acclimation, the lower abdominal region of the mice was subjected to stimulation using six filaments, each marked with distinct pressure levels. Care was taken to avoid repetitive irritation of the same area in order to prevent desensitization. Each mouse underwent a series of 10 tests, and the outcomes were represented as respective percentages. Three categories of behaviour were classified as indicative of positive responses: (1) sudden retraction of the abdomen; (2) licking or scratching of the region receiving filament stimulation; (3) jumping.^22^

### 2.3 Histopathological examination

The prostate was obtained from the mice, preserved in 4% neutral formalin for at least 1 week, and subsequently embedded in paraffin wax. The paraffin‐embedded tissue was sectioned to a thickness of 5 μm and stained by haematoxylin and eosin. The stained slides were then scanned using a digital slide scanner (KFBIO; KF‐PRO‐005‐EX) and evaluated. The degree of inflammation was assessed using defined quantitative criteria: 0, no inflammation; 1, mild perivascular cuffing with mononuclear cells; 2, moderate perivascular cuffing with mononuclear cells; 3, marked perivascular cuffing, haemorrhage and the presence of numerous mononuclear cells within the parenchyma.^21^

### 2.4 Immunohistochemistry

The paraffin‐embedded tissue slides were deparaffinized and then subjected to antigen retrieval by heating in citric acid buffer (10 mM, pH 6.0; Servicebio, G1202). The SABC‐HRP Kit (Beyotime Biotechnology Co. Ltd, P0615) was employed for the subsequent experimental procedures, following the producer's instructions. The primary antibody employed was as follows: anti‐CCL20 antibody (1:200; Affinity, DF2238) and anti‐IL‐17A antibody (1:200; Affinity, DF6127). The proceeded slices were scanned using a digital slide scanner (KFBIO; KF‐PRO‐005‐EX). The relative expression levels were quantified using IHC Profiler,^25^ a plugin of ImageJ (National Institutes of Health, Bethesda, MD).

### 2.5 Immunofluorescence

The paraffin‐embedded tissue slides were dewaxing and antigen retrieval utilizing citric acid buffer (10 mM, pH 6.0; Servicebio, G1202). Following this, block the glass slides with BSA and incubate overnight at 4°C with the specified primary antibody. The primary antibody employed was as follow: anti‐CCL20 antibody (1:200; Affinity, DF2238); anti‐CD4 antibody (1:500; Elabscience, E‐AB‐22098); anti‐CD116 antibody (1:500; Affinity, DF4820); anti‐alpha SMA (α‐SMA) antibody (1:500; Affinity, BF9212); anti‐vimatin antibody (1:500; Affinity, BF8006). Subsequently, the slides were left at room temperature for 2 h with the appropriate secondary antibodies, which were as follows: Cy3‐conjugated Goat Anti‐Rabbit IgG (1:500; Servicebio, GB21303); FITC‐conjugated Goat Anti‐Mouse IgG (1:200; Servicebio, GB22301). The marked slides were always protected from light and were dyed using DAPI (Servicebio; G1012). The co‐localization of CCL20 with other biomarkers was recorded using confocal laser scanning microscopy (Olympus; FV3000). The fluorescence signal was quantified using ImageJ.

### 2.6 Elisa assay

The cytokines levels in serum of mice were measured by employing the Elisa kit for IL‐17A (Elabscience, E‐EL‐M0047c), IL‐17F (Multi‐Science, EK2196), IL‐22 (Elabscience, E‐EL‐M2446), IFN‐γ (Elabscience, E‐EL‐M0048), TNF‐α (Elabscience, E‐EL‐M3063), GM‐CSF (Elabscience, E‐MSEL‐M0022) and CCL20 (Elabscience, E‐EL‐M0013). All procedures were carried out under the producer's instructions.

### 2.7 RNA isolation and RT‐qPCR

Total RNA was extracted using the RNA purification kit plus (Esscience, RN002plus). The quality and content of the extracted RNA were determined using a spectrophotometer (NanoDrop 2000). Reverse transcription and genomic DNA cleanup were conducted with PrimeScript™ RT reagent Kit (Takara, RR047A), and no dilution of the resulting product took place. RT‐qPCR was conducted using TB Green® Premix Ex (Takara, RR820A) with a reaction volume of 20 μL. The reactions were recorded using the ABI7500 platform (Thermo, MA, USA). Data analysis was performed using the 2^−ΔΔCt^ method. All samples were assessed in triplicate to minimize procedural errors. The primers used were synthesized by Sangon Biotech (Sangon, Shanghai, China), and the sequence was listed in the Table S1. In this section, we followed the official protocol for execution.

### 2.8 Flow cytometry

Single‐cell suspensions of spleens were harvested by homogenizing the mouse spleen tissue and subsequently passing it through a 400‐mesh sieve. Single‐cell suspension of the prostate was prepared by digestion of minced mouse prostate tissue. The digestion solution was formulated using RPMI‐1640 (BasalMedia, L210KJ) supplemented with FBS (10%; WISENT Corporation, 086–150), DNase I (0.01%; Roche, #10104159001), HEPES (10 mM; Gibco, #15630106) and collagenase D (1 mg/mL; Roche, #11088858001). APC‐anti‐CD3e (BD Bioscience, 553,066), FITC‐anti‐CD4 (BD Bioscience, 553,047), PE‐anti‐IL‐17A (BD Bioscience, 559,502) and PerCP‐Cy‐anti‐F4/80 (BD Bioscience, 746,070) were used. The surface markers were stained by incubating with fluorescence‐conjugated antibodies at 4°C for 1 h. For intracellular cytokine staining, cells were stimulated using a complete RPMI‐1640 containing PMA (50 ng/mL; Multi‐Science, CS0001), ionomycin (1000 ng/mL; Multi‐Science, CS0002) and monensin (25 μg/mL; Multi‐Science; CS0004) for 4 h at 37°C, 5% CO2. Subsequently, cells were fixed and permeabilized using a fixation/permeabilization buffer (Invitrogen, 00‐5523‐00). A CytoFLEX flow cytometer (Beckman Coulter, Brea, CA) was used to record the stained cells, and the data were analysed using FlowJo_v10.6.2 (Tree Star, Ashland).

### 2.9 Western blot

Total protein was prepared by extracting the supernatant after lysing tissues or cells with RIPA lysis buffer (Elabscience, E‐BC‐R327) and subsequently adding a loading buffer (wshtbio, ES003). The proteins were resolved using a 12.5% SDS‐PAGE gel (Epizyme, Shanghai, China) and subsequently blotted onto 0.22 μm nitrocellulose membranes (Millipore, America). After incubation with 5% BSA for 1 hour at room temperature, the membrane was incubated with primary antibody overnight at 4°C. The primary antibodies used were as follows: anti‐GAPDH (1:1000; Elabscience, E‐AB‐40337), anti‐CCR6 (1:1000; SinoBiogical, #106771‐T32), anti‐IL‐17A (1:1000; Affinity, DF6127), anti‐CCL20 (1:1000; Affinity, DF2238), anti‐P65 (1:1000, Affinity, AF5006), anti‐Phospho‐P65 (1:1000, Affinity, AF2006), anti‐ERK1/2 (AF0155), anti‐Phospho‐ERK1/2 (1:1000, Affinity, AF1015), anti‐P38 (1:1000, Affinity, AF6456), anti‐Phospho‐P38 (1:1000, Affinity, AF4001), anti‐mTOR (1:1000, CST, 2983 T), anti‐Phospho‐mTOR (1:1000, CST, 5536 T), anti‐AKT (1:1000, CST, 4691 T) and anti‐Phospho‐AKT (1:1000, CST, 4060 T). Then the bands were incubated with the secondary antibodies (1:5000; Elabscience, E‐AB‐1003), which was derived from rabbit and conjugated with HRP. Subsequently, blots were captured with an ECL luminescence system (ClinX, Shanghai, China). The gels were quantified and analysed using ImageJ.

### 2.10 Cell culture

Primary Th17 cells, bone marrow‐derived macrophages (BMDM), and the RAW264.7 cell line were employed to investigate the findings. They were all cultured in an incubator at 37°C with 5% CO_2_. Th17 and BMDM were cultured in RPMI‐1640 containing 10% FBS and 1% Penicillin–Streptomycin Solution. RAW264.7 cells were cultured in a high‐glucose DMEM medium supplemented with 10% FBS and 1% Penicillin–Streptomycin Solution. Th17 and BMDM are non‐proliferative or have low proliferation rates and were collected before each experiment. RAW264.7 has the ability to proliferate and was passaged every 2 days at a ratio of 1:5. The RAW264.7 cells were frozen every other generation. The freezing medium consisted of high‐glucose DMEM culture medium, FBS and DMSO in a ratio of 5:4.5:0.5. After placing the cells in a programmed freezing container, they were initially stored in a − 80°C freezer overnight before being transferred to liquid nitrogen.

BMDM and RAW264.7 cells required drug treatment in a serum‐free culture medium before the experiments. They were incubated with IL‐17A (100 ng/mL; Peprotech, 210–17) for 12 h, followed by media replacement with a serum‐free culture medium. After that, they were incubated with LPS (100 ng/mL; Solarbio, L8880) for 12 h and either total protein or total RNA was extracted.

### 2.11 Primary cell isolation and differentiation

A clean biological safety cabinet is essential as it effectively prevents bacterial contamination. NOD mice were euthanized by cervical dislocation under lidocaine anaesthesia. Subsequently, the mice were immersed in 75% ethanol for 5 min for disinfection before being transferred to a sterile laminar flow hood for further procedures on a sterile surgical drape. Naive CD4^+^ T cells (CD4^+^CD25^−^CD44^Low^CD62L^High^) were isolated from a single‐cell suspension extracted mouse spleen.^22^ Subsequently, they were transferred to 6‐well plates that had been pre‐coated with anti‐CD3 and anti‐CD28 antibodies. The collected naive CD4^+^ cells had a purity exceeding 95% (Figure S1A). After 4 days of cultivation in RPMI‐1640 containing 10% FBS and supplemented with IL‐6 (40 ng/mL; Novoprotein, CG39), IL‐23 (40 ng/mL; Novoprotein, CS31), hTGF‐β (1 ng/mL; Novoprotein, CA59), anti‐IFN‐γ (20 μg/mL; Bio X Cell, BE0055) and anti‐IL‐4 (20 μg/mL; Bio X Cell, BE0045), a certain proportion of Th17 cells could be obtained (Figure S1B).

The tibia and femur of the mice were carefully isolated, and the attached muscles were removed. Subsequently, the bone ends were snipped open using ophthalmic scissors, and the bone marrow was flushed out with cold PBS. After red blood cell lysis, the collected cells were transferred to a 6‐well plate. The cells were cultured in RPMI‐1640 containing 10% FBS and M‐CSF (50 ng/mL; Novoprotein, CB34) for 7 days, with medium changes every 3 days. The purity of BMDM was shown (Figure S1C).

### 2.12 Cell viability test

The cell counting kit‐8 (CCK‐8; Servicebio, G4103) was employed to assess cell viability. 5 × 10^3^ Th17 cells were seeded into a 96‐well plate, with the addition or absence of rCCL20 or the anti‐CCL20 neutralizing antibody. After 12 h of incubation, the CCK‐8 detection reagent was added and left to incubate for a duration of 2 h. The relative cell viability was calculated based on the OD values measured at 450 nm.

### 2.13 Transwell

The transwell assay was conducted using a 24‐well plate with 8 μm pore‐size chambers to assess the impact of CCL20 on the migration of Th17 cells. Each chamber was filled with 200 μL of serum‐free high‐glucose DMEM medium, containing approximately 1 × 10^4^ primary Th17 cells that were suspended in the medium. The bottom of the 24‐well plate was loaded with 600 μL of high‐glucose DMEM containing 10% FBS, with or without rCCL20 (10 ng/mL; Peprotech, 250–27) or anti‐CCL20 neutralizing antibody (10 ng/mL; R&D system, MAB421). After incubating for 12 h at 37°C with 5% CO_2_, the cells in the lower chamber were counted, and the relative number of migrated cells was calculated using the control group as a reference.

### 2.14 Statistical analysis

Statistical analysis was carried out utilizing GraphPad Prism 9 (La Jolla, CA, USA). All experiments were independently replicated a minimum of three times. Data were presented as the mean ± standard deviation (SD). Statistical analysis was conducted using either a two‐tailed Student's *t*‐test or a one‐way analysis of variance (anova) followed by the Bonferroni post hoc test. Significance was considered at a level of *p* < 0.05. In the figures, the string (N.D.) represented undetermined, and asterisks (*), double asterisks (**), and triple asterisks (***) were used to denote statistical significance at *p* < 0.05, *p* < 0.01, and *p* < 0.001, respectively.

## 3 RESULTS

### 3.1 Excessive activation of CD4 ^+^ IL‐17A ^+^ cells in the spleen of EAP mice

**FIGURE 1:**
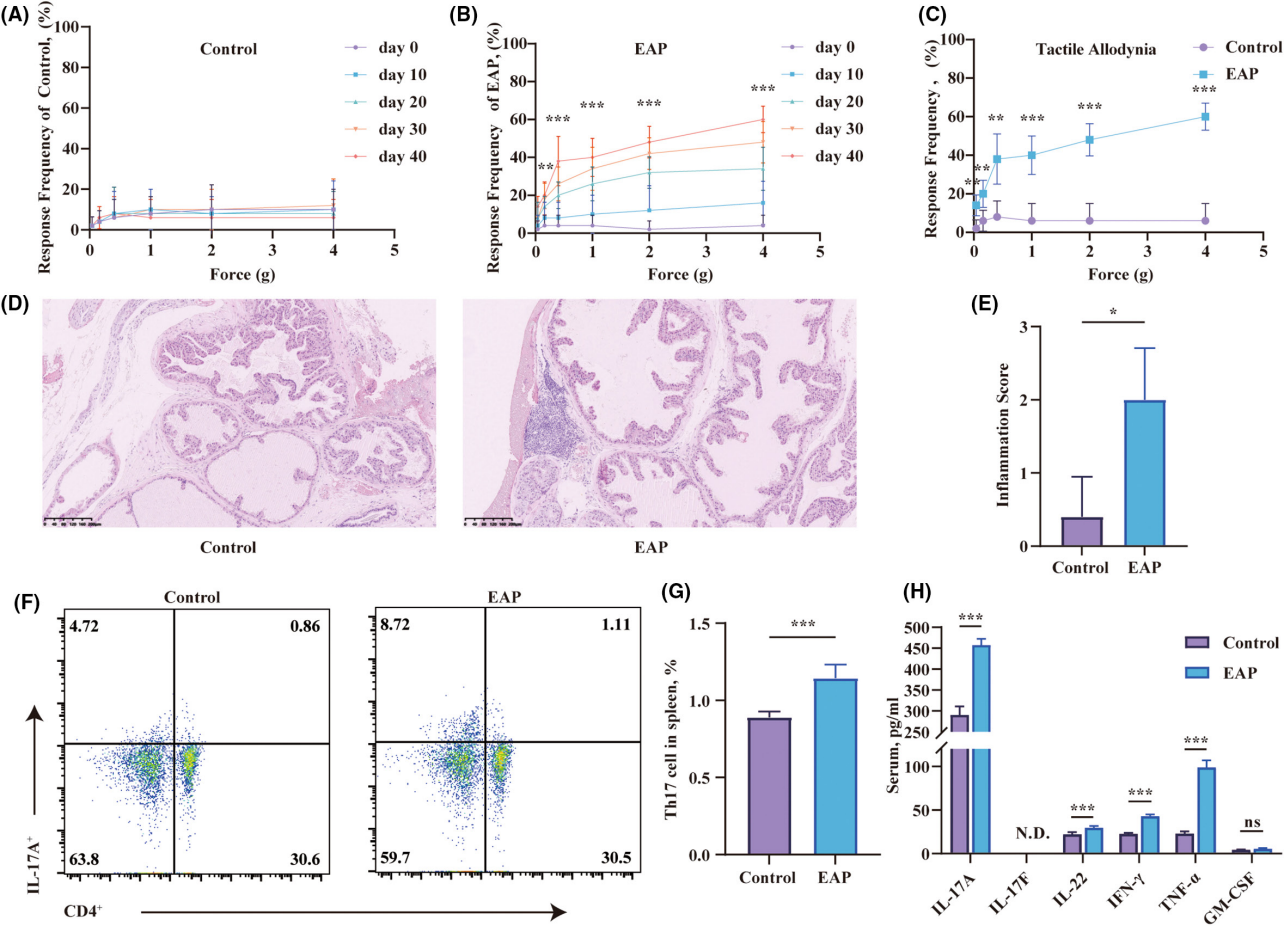
Experimental autoimmune prostatitis (EAP) induction led to pelvic discomfort, histological alterations in prostate tissues, heightened activity of CD4 + IL‐17A+ cells in splenic lymphocytes and elevated levels of IL‐17 in the serum. (A–C) Pelvic pain induced by EAP was evaluated through von Frey testing for tactile allodynia in NOD mice. This involved measuring the response frequencies to mechanical filament stimulation of the pelvic region. (D) Representative images of HE staining. (E) Histological assessment to determine the extent of inflammation. (F, G) Flow cytometry staining of splenic lymphocytes to detect CD4 + IL‐17A+ cells. (H) Quantitative analysis of inflammatory cytokines (IL‐17A, IL‐17F, IL‐22, IFN‐γ, TNF‐α and GM‐CSF) in the serum of mice determined by ELISA. Representative data from three independent experiments are shown. Data were presented as mean ± SD and were analysed using one‐way anova analysis (A–C), unpaired, two‐tailed Student's *t*‐test analysis (G, H) or Wilcoxon rank‐sum test (E). ‘N.D.’ Not Determined; *p < 0.05; **p < 0.01; ***p < 0.001.

The EAP model was proficiently induced by administering mixtures of PAgs and CFA through subcutaneous injections on days 0 and 28. The effectiveness of the EAP model was evaluated using the Von Frey filaments test and haematoxylin and eosin staining. The Von Frey test was conducted on the mice every 10 days following immunization. The control group showed no substantial rise in pain frequency (Figure 1A). In contrast, the EAP mice showed a strong correlation between pain frequency and time as well as the applied force (Figure 1B). EAP mice displayed heightened stress responses, regardless of whether it was due to the passage of time or increasing levels of applied forces. On the 40th day post‐immunization, the EAP group displayed more pronounced tactile allodynia in the lower abdomen area compared to the control group (Figure 1C). Significant infiltration of inflammatory cells was also observed in the prostate of EAP mice (Figure 1D). There were more cells with enlarged nuclei and intense staining in the prostate of EAP mice. Based on the assessment of the location of these infiltrating cells, the inflammation score in the EAP group was significantly higher than that in the control group (Figure 1E). Th17 cells, with their IL‐17A secretion, were central players in autoimmune disease research. To explore whether Th17 cells affected the development of EAP, flow cytometry and ELISA were performed. The spleen of the EAP group showed a higher activation of CD4^+^IL‐17A^+^ cells compared to the control group (Figure 1F,G). Serum cytokine analysis revealed elevated levels of pro‐inflammatory cytokines (IL‐17A, IL‐22, IFN‐γ, and TNF‐α) in the EAP group compared to controls (Figure 1H). However, IL‐17F was not determined, and GM‐CSF levels remained comparable between the groups.

### 3.2 Enhanced CD4 ^+^ IL‐17A ^+^ cell proportions in the prostate of EAP mice

**FIGURE 2:**
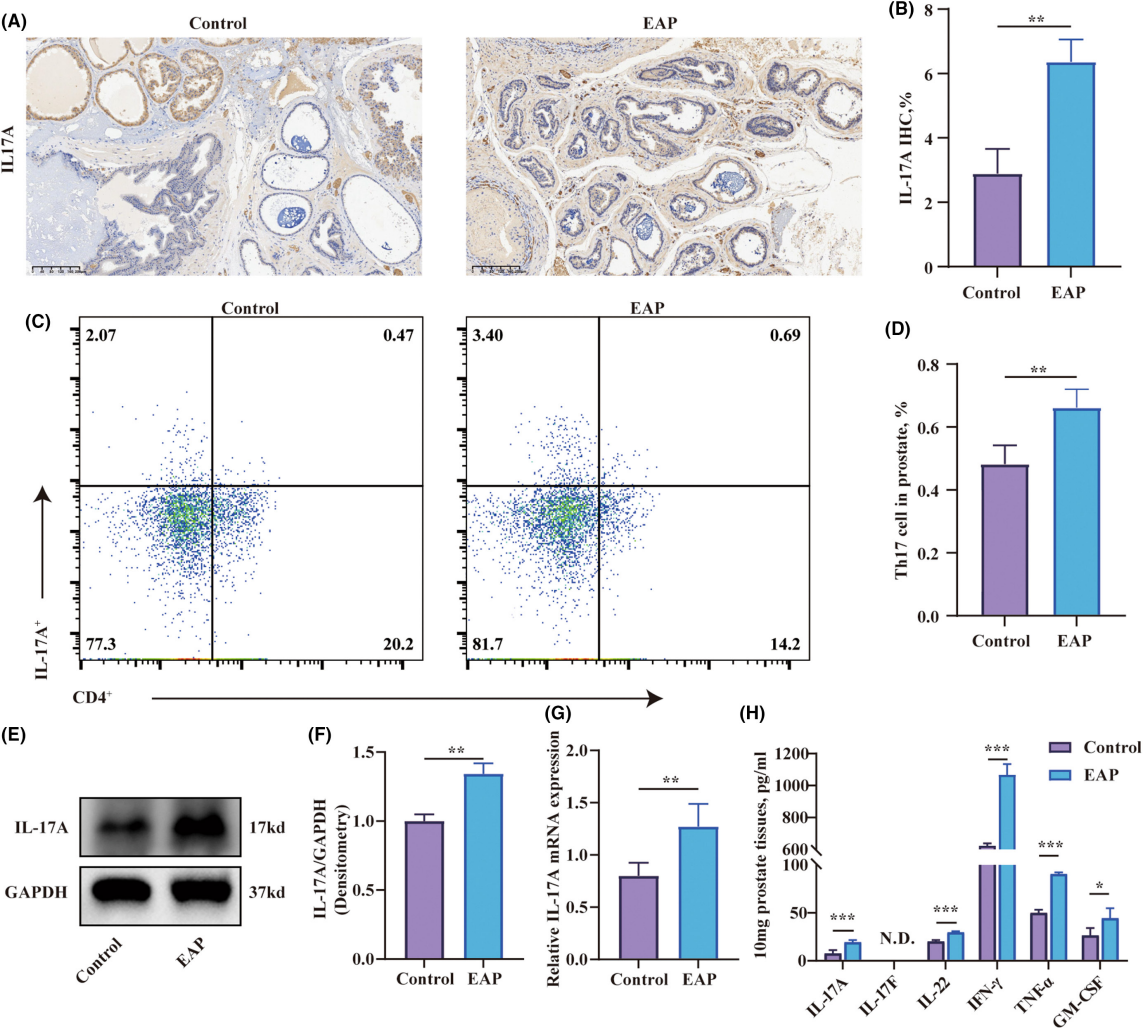
The increased infiltration of Th17 cells in the prostate in experimental autoimmune prostatitis mice. (A) Immunohistochemical staining of IL‐17A (marker of Th17) on paraffin‐embedded prostate sections. (B) Semi‐quantitative analysis of IL‐17A in Immunohistochemical staining. (C, D) Flow cytometry analysis of CD4 + IL‐17A+ cells in prostate tissues. (E, F) The IL‐17A expression in prostate tissues assessed through Western Blot analysis. (G) Relative expression of IL‐17A in the prostate measured through RT‐qPCR. (H) Quantitative analysis of inflammatory cytokines (IL‐17A, IL‐17F, IL‐22, IFN‐γ, TNF‐α, GM‐CSF) in the prostate of mice determined by ELISA. Representative data from three independent experiments are shown. Data were presented as mean ± SD and were analysed using unpaired, two‐tailed Student's *t*‐test analysis. ‘N.D.’ Not Determined; *p < 0.05; **p < 0.01; ***p < 0.001.

The above findings have confirmed the overactivation of Th17 cells in the spleen, consistent with the findings reported by Zhan et al.^22^ Next, we aimed to validate whether Th17 cells were being recruited to the prostate of EAP mice, thus influencing the progression of EAP. The results of immunohistochemistry indicated that the EAP group had a higher number of IL‐17A‐stained cells in the prostate compared to the control group (Figure 2A,B). Flow cytometry was employed to measure the change in the percentage of Th17 cells in the prostate. The result suggested a significant increase in the percentage of CD4^+^IL‐17A^+^ cells in the prostate of the EAP group compared to the that of control group (Figure 2C,D). Moreover, the transcription and expression levels of IL‐17A in the EAP group's prostate were also elevated compared to the control group (Figure 2E–G). Prostate from EAP mice exhibited elevated concentrations of IL‐17A, IL‐22, IFN‐γ, TNF‐α and GM‐CSF compared to controls (Figure 2H). The measurement of IL‐17A levels and CD4^+^IL‐17A^+^ cells confirmed the potential association of Th17 with EAP.

### 3.3 CCL20 was overexpressed in the prostate of EAP mice

**FIGURE 3:**
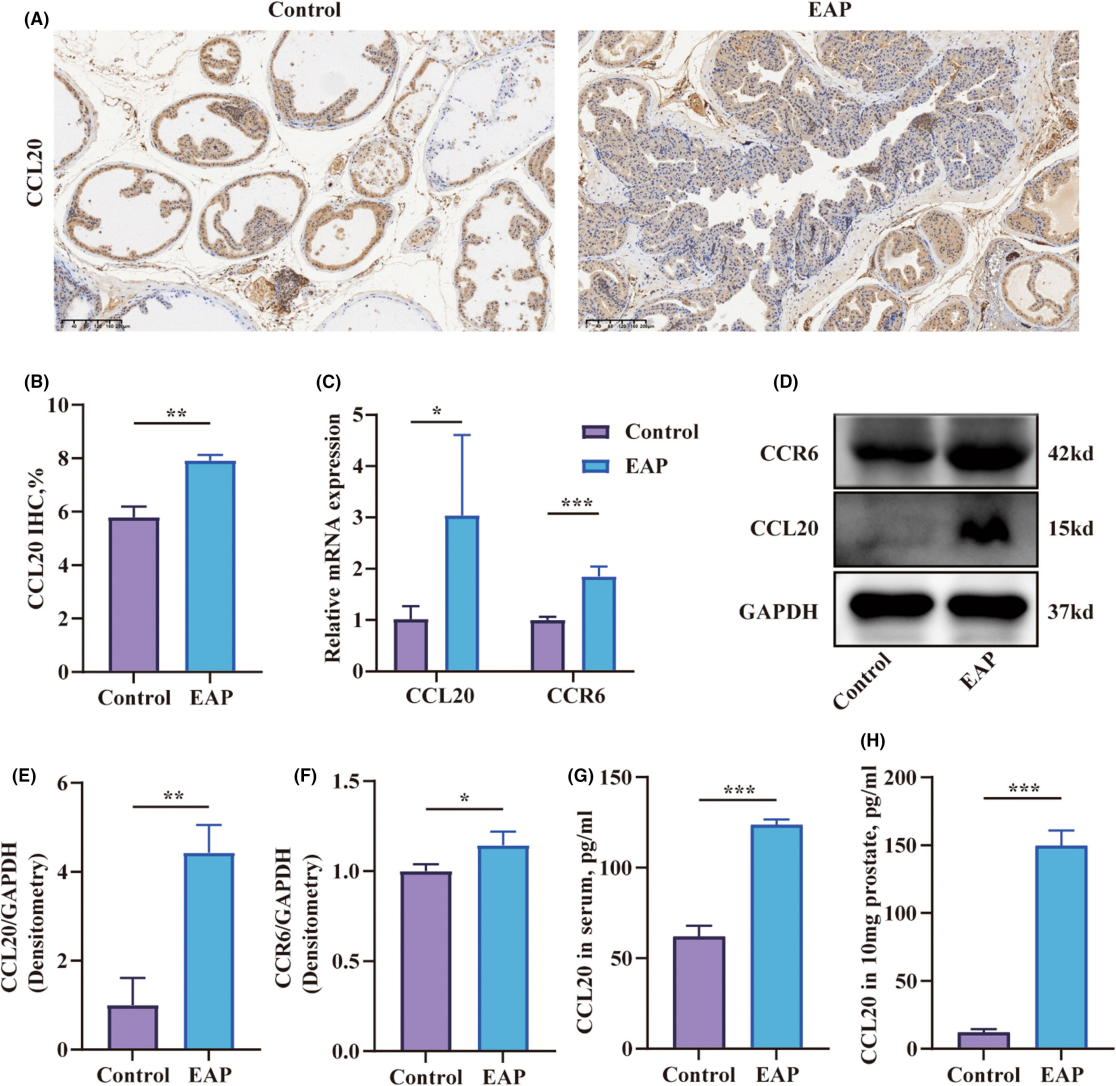
The increased levels of CCL20 in the prostate in experimental autoimmune prostatitis mice. (A) Immunohistochemical staining of CCL20 on paraffin‐embedded prostate sections. (B) Semi‐quantitative analysis of CCL20 in Immunohistochemical staining. (C) Relative expression of CCL20 and CCR6 in the prostate measured by RT‐qPCR. (D–F) The expression level of CCL20 and CCR6 in the prostate tissues assessed by Western Blot. (G, H) Quantitative analysis of CCL20 in the serum and prostate of mice determined by ELISA. Representative data from three independent experiments are shown. Data were presented as mean ± SD and were analysed using unpaired, two‐tailed Student's t‐test analysis. *p < 0.05; **p < 0.01; ***p < 0.001.

The infiltration of Th17 cells in the prostate of EAP mice raised the question of whether there was a specific cytokine that could selectively attract Th17 cells to the prostate. Chemokines, a class of proteins with a molecular weight of approximately 8–10 kd, have a significant role in inflammation by attracting white blood cells to migrate to the site of infection. The chemotaxis family consists of CC chemokines, CXC chemokines, C chemokines, and CX3C chemokines, and they mediate chemotactic responses by binding to their corresponding receptors. Although CCR6 is selectively expressed in immature DCs, effector or memory CD4^+^ T lymphocytes and B lymphocytes, it is stably expressed on the membrane of Th17 cells.^13^ The expression of CCL20, the exclusive ligand of CCR6, in the prostate of EAP mice, became a subject of inquiry. The results of immunohistochemistry confirmed that CCL20 positive‐staining cells were increased in the prostate tissues of EAP mice (Figure 3A,B). Furthermore, the transcription levels of CCL20 and CCR6 in the prostate of the EAP group were elevated compared to the control group (Figure 3C). The cytokines levels of CCL20 and CCR6 also showed corresponding increases (Figure 3D–F). Serum and prostate CCL20 levels were elevated in the EAP group relative to controls (Figure 3G,H).

### 3.4 Th17 was recruited by prostate‐secreted CCL20

**FIGURE 4:**
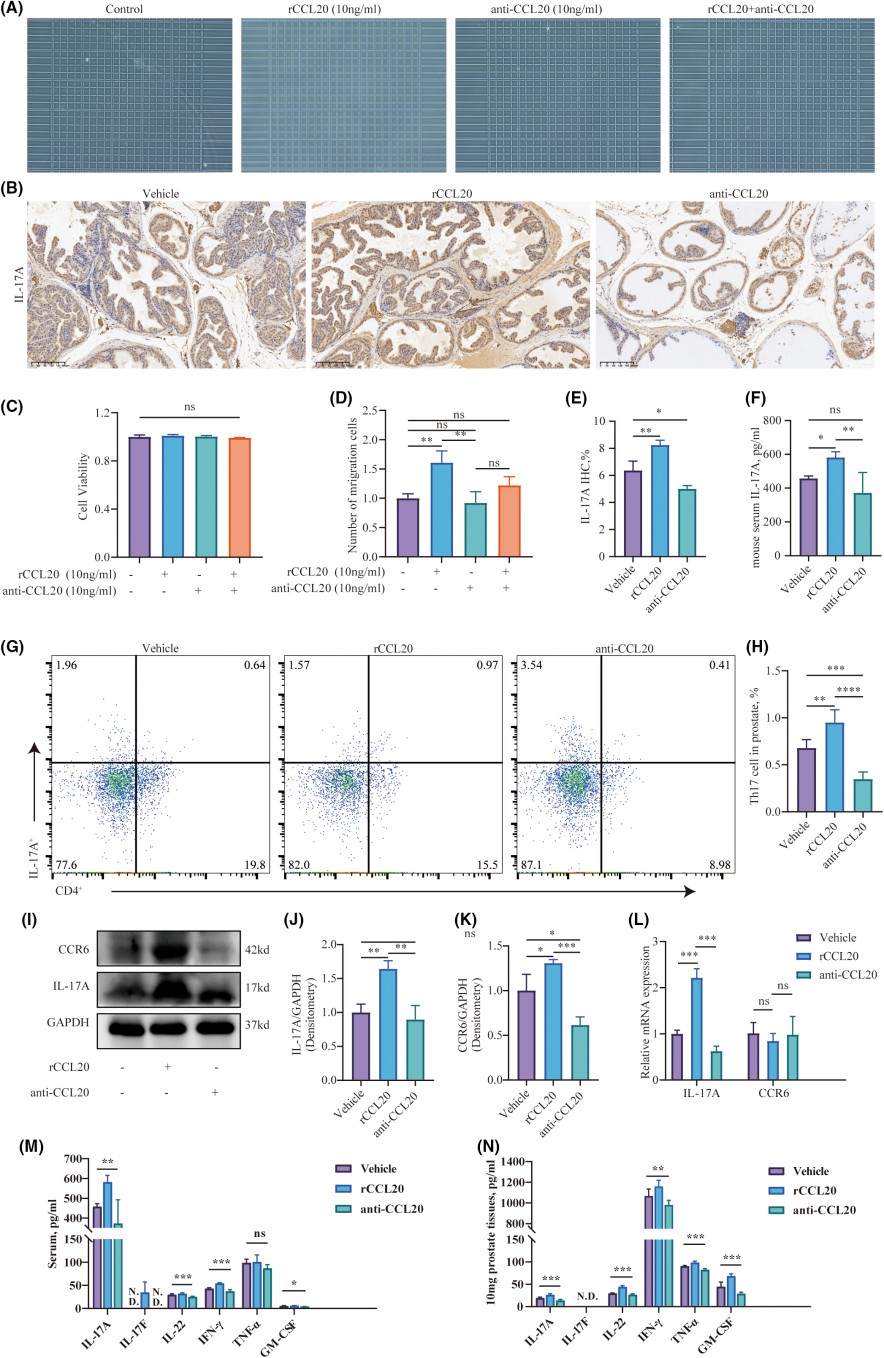
The recruitment of Th17 cells by CCL20. (A, D) Counting Th17 cells chemoattracted by CCL20 through transwell assay. (B) Immunohistochemical staining of IL‐17A on paraffin‐embedded prostate sections in each group. (C) Assessment of cell viability of Th17 cells after treatment with rCCL20 or anti‐CCL20 neutralizing antibodies. (E) Semi‐quantitative analysis of CCL20 in Immunohistochemical staining. (F) Quantitative analysis of IL‐17A in the serum of mice determined by ELISA. (G, H) Flow cytometry analysis of CD4^+^IL‐17A^+^ cells in the prostate. (I–K) The expression level of IL‐17A and CCR6 in the prostate tissues determined by Western Blot. (L) Relative expression of IL‐17A and CCR6 in the prostate measured by RT‐qPCR. Representative data from three independent experiments are shown. (M, N) Quantitative analysis of inflammatory cytokines (IL‐17A, IL‐17F, IL‐22, IFN‐γ, TNF‐α, GM‐CSF) in the serum and prostate of mice determined by ELISA. Data were presented as mean ± SD and were analysed using one‐way anova analysis. ‘N.D.’ Not Determined; ‘ns’ *p* > 0.05; *p < 0.05; **p < 0.01; ***p < 0.001.

To investigate whether the recruitment of Th17 cells was linked to the elevated levels of CCL20 in the prostate of EAP mice, we examined the effects of rCCL20 and anti‐CCL20 neutralizing antibodies on Th17 cell migration in vitro. The results demonstrated that neither rCCL20 nor the anti‐CCL20 neutralizing antibody had any effect on the viability of Th17 cells (Figure 4C). In the Th17 migration assay, the rCCL20 group exhibited an increased chemotaxis of Th17 cells compared to the control group. The anti‐CCL20 antibody group didn't show significant chemotaxis inhibition. Notably, the anti‐CCL20 neutralizing antibody was capable of inhibiting the chemotactic effect induced by rCCL20 (Figure 4A,D). This might be attributed to the absence of endogenous CCL20 in the in vitro environment.

Subsequently, we studied the impact of rCCL20 and anti‐CCL20 neutralizing antibodies on the chemotaxis of Th17 cells at non‐toxic concentrations in vivo. We evaluated alterations in IL‐17A levels in the prostate and serum of EAP mice by intraperitoneally injecting either rCCL20 or anti‐CCL20 neutralizing antibody. The results of Immunohistochemistry staining showed the levels of IL‐17A were positively correlated with the levels of CCL20 (Figure 4B,E). The group receiving rCCL20 injections showed a higher count of IL‐17A positive‐staining cells in their prostate glands compared to the vehicle group. Conversely, mice injected intraperitoneally with anti‐CCL20 antibodies exhibited a decrease in IL‐17A positive‐staining cells in their prostates compared to the vehicle group. A corresponding change in the concentration of IL‐17A in the serum was also observed after the injection of rCCL20 or the anti‐CCL20 neutralizing antibody (Figure 4F). Additionally, the proportion of CD4^+^IL‐17A^+^ cells in the prostate was increased by rCCL20 injection and decreased by the anti‐CCL20 neutralizing antibody (Figure 4G,H). The assessment of cytokines within the prostate substantiated this observation (Figure 4I–K). Consistently, the transcription levels of IL‐17A exhibited the same alterations following the injection of rCCL20 or anti‐CCL20 neutralizing antibody (Figure 4L). Furthermore, following administration of rIL‐17A or anti‐IL‐17A neutralizing antibody, inflammatory cytokines in serum and prostate underwent changes (Figure 4M,N).

### 3.5 Stromal cells of the prostate and immune cells were potential sources of CCL20

**FIGURE 5:**
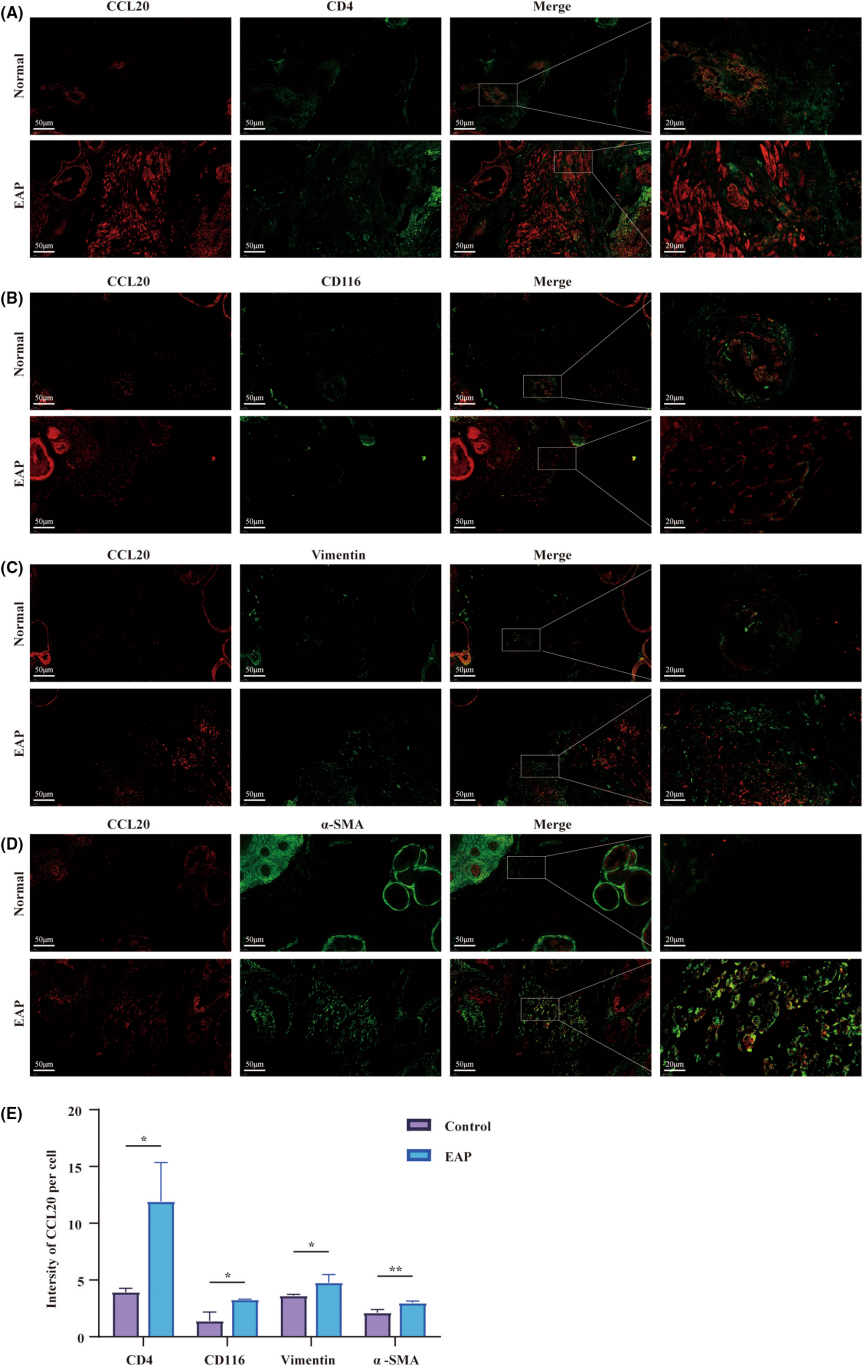
The potential source CCL20 in the prostate of experimental autoimmune prostatitis (EAP) mice. Representative photographs of immunofluorescence staining for CCL20 and markers for (A) CD4^+^ T cells, (B) macrophages and (C, D) prostatic stromal cells of EAP mice. (E) Quantification of CCL20 immunofluorescence intensity in the experiments of (A–D). Representative data from three independent experiments are shown. Data were presented as mean ± SD and were analysed using one‐way anova analysis. *p < 0.05; **p < 0.01; ***p < 0.001.

Immunohistochemical analysis reveals the presence of cells capable of expressing and secreting CCL20 within the murine prostate (Figure 3A). To investigate the exact source of CCL20, we conducted dual‐colour immunofluorescence co‐localization analysis. CCL20 and phenotypic cell markers were labelled with different antibodies and fluorescent conjugates for analysis (Red represents CCL20; Green represents phenotypic markers: CD4 for CD4^+^ T cells, CD116 for macrophage, α‐SMA and Vimentin for stromal cells). The localization of CCL20 overlapped with the localization of CD4^+^ T cells (Figure 5A), macrophage (Figure 5B) and stromal cells (Figure 5C,D). We quantified and compared the mean fluorescence intensity of CCL20 within each cell type (Figure 5E). Higher fluorescence intensity of CCL20 was observed in CD4^+^ T cells, macrophages and stromal cells in the EAP group compared to the control group, which was consistent with the conclusions drawn in previous papers. Yamazaki et al. suggested that Th17 cells could also secret CCL20, the sole ligand of CCR6, in addition to expressing CCR6.^26^ Walch‐Rückheim et al.^27^ hinted that IL‐6 induced the regulator of CCL20 in fibroblasts via the C/EBPβ pathway. Liu et al. also reported that IL‐4 promotes M2‐type macrophages to express CCL20.^28^ All these hinted stromal cells of the prostate and immune cells might potentially serve as a source of CCL20 in EAP mice.

### 3.6 IL‐17A stimulated macrophage to secret CCL20 via the NF‐ΚB pathway

**FIGURE 6:**
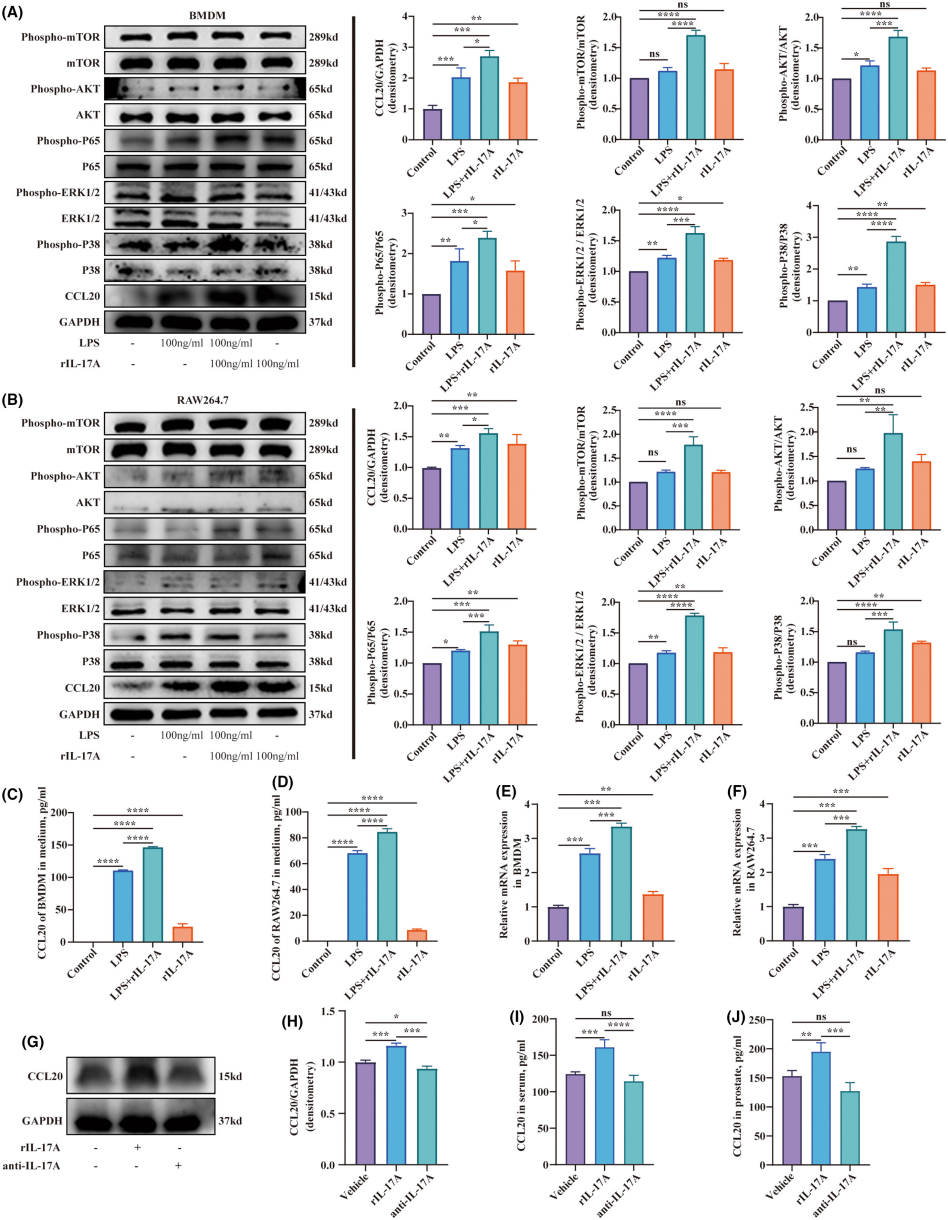
IL‐17A promoted secretion of CCL20 of macrophages via the NF‐κB/MAPK/PI3K pathway. (A, B) Western blot analysis of CCL20 and the phosphorylation of the PI3K (mTOR and AKT) NF‐κB (P65) and MAPK (ERK1/2, P38) pathways in BMDM and RAW264.7 after treatment of LPS and/or rIL‐17. (C, D) Quantitative analysis of CCL20 in the supernatant of BMDM and RAW264.7 determined by ELISA. (E, F) Relative expression of CCL20 in BMDM and RAW264.7 measured by RT‐qPCR after treatment of LPS and/or rIL‐17A. (G, H) Western blot analysis of CCL20 after injection of rIL‐17A or anti‐IL‐17A neutralizing antibodies. (I, J) Quantitative analysis of CCL20 in the serum and prostate of mice determined by ELISA. Representative data from three independent experiments are shown. Data were presented as mean ± SD and were analysed using one‐way anova analysis. ‘ns’ *p* > 0.05; *p < 0.05; **p < 0.01; ***p < 0.001.

To explore the mechanism of CCL20 secreting in the prostate, experiments involving macrophages were conducted. Macrophages, one of the antigen‐presenting cells, are easily accessible for primary cell extraction and possess various cell lineages. BMDM, which stands for bone marrow‐derived macrophage, are primary cells obtained through the differentiation of bone marrow haematopoietic stem cells in vitro. They were often utilized in immunological research. RAW264.7, a macrophage cell line, was established from leukaemia cells in Abelson mice. These two kinds of cells were employed to explore the impact of IL‐17A in CCL20 secreting. They were induced into an inflammatory state using LPS, and then either treated with or without the exogenous cytokine of IL‐17A. Furthermore, IL‐17A was also added alone to explore whether it affects the normal macrophage. The results showed that BMDM in an inflammatory state could secret more CCL20 than the control group, and IL‐17A further upregulated the secreting of CCL20. Additionally, BMDM treated with IL‐17A expressed more CCL20 than the control group (Figure 6A). The phosphorylation level of mTOR, AKT, p65, ERK1/2 and P38 also exhibited similar changes (Figure 6A). RAW264.7 cells stimulated by LPS expressed more CCL20 compared to the control group. The addition of IL‐17A significantly increased CCL20 expression. Furthermore, the group cultured only with IL‐17A solely exhibited increased CCL20 expression compared in comparison to controls (Figure 6B). Phosphorylation of mTOR, AKT, p65, ERK1/2 and P38 in RAW264.7 exhibited similar alterations (Figure 6B). Consistently, the CCL20 concentration in the supernatant and transcript level in BMDM and RAW264.7 cells displayed parallel changes (Figure 6C–F). We also investigated the changes of CCL20 levels in the prostate in EAP mice after intraperitoneal injection of rIL‐17A or anti‐IL‐17A neutralizing antibody. The results showed that mice treated with rIL‐17A had higher CCL20 expression compared to the vehicle group, while mice injected with anti‐IL‐17A neutralizing antibody showed a decrease in CCL20 expression (Figure 6G,H). The measurement of CCL20 in both serum and prostate using Elisa kits demonstrated similar changes (Figure 6I,J).

## 4 DISCUSSION

CP/CPPS, a prevalent malady in urology, is a syndrome characterized by pelvic distress, persisting for a minimum of 3 months within the preceding 6 months. It often co‐occurs with urological symptoms and/or sexual dysfunction.^1^ CP/CPPS have a substantial influence on the quality of life among adult males. Due to a limited understanding of its pathophysiology, the diagnosis and treatment of CP/CPPS are unreliable.^1^, ^29^

Enhancing the management of CP/CPPS had led tof the exploration of various hypotheses, such as cryptic infections, oxidative stress, autoimmune mechanisms and abnormal pelvic floor neuromuscular activity.^4^ Th17 cells, a subset of T helper cells with pro‐inflammatory properties, have been implicated in various autoimmune disease.^5^, ^6^ IL‐17A, a cytokine produced by Th17 cells, induced inflammation through mediating IL‐6 production and neutrophil recruitment.^5^ Motrich et al.^30^ observed increasing levels of IL‐17A in the serum and seminal plasma of patients with CP/CPPS. The EAP model, an animal model constructed for the investigation of the immune mechanisms of CP/CPPS, has garnered favour among researchers.^21^, ^22^, ^31^ Zhan et al.^22^ showed excessive activation of Th17 cells and elevated IL‐17A in the prostate of EAP mice. Nevertheless, the mechanism responsible for recruiting Th17 cells to the prostate remains elusive. As previous paper, Th17 cells could be recruited to the inflammatory site via the CCL20/CCR6 axis.^18^, ^19^, ^20^ Hua et al.^21^ confirmed the ability of stromal cells and immune cells in the mouse prostate to secrete chemotactic cytokines. Liu et al. suggested that stromal cells in the inflammatory site could produce CCL20.^26^, ^27^, ^28^

In this study, the increase of IL‐17A was confirmed in the serum and prostate of EAP mice. The increasing proportion of Th17 cells in the prostate of EAP mice was also demonstrated through flow cytometry analysis. These are consistent with the conclusion drawn by Zhan et al.^22^ Furthermore, this study established a connection between CCL20 and the Th17 cells homing to the prostate. CCL20 is the sole ligand of CCR6, a receptor stably expressed on the membrane of Th17 cells.^26^ Primary Th17 cells exhibit chemotaxis in response to exogenous CCL20 in vitro. This study also confirmed that the increase in CCL20 might exacerbate EAP, while anti‐CCL20 neutralizing antibodies could alleviate EAP.

To explore whether stromal cells or immune cells in the prostate produced CCL20, an immunofluorescence co‐localization assay was performed. It revealed that CCL20 exhibited colocalization with stromal cells and immune cells, consistent with the findings of Hua et al.'s study.^21^ Moreover, the IL‐17A secreted by the recruited Th17 cells might promote macrophages to produce CCL20 via the NF‐κB/MAPK/PI3K pathway. From these observations, it becomes evident that a reinforcing, positive feedback loop exists between CCL20 and IL‐17A, thereby amplifying the severity of EAP.

In summary, this study provides some shreds of evidence linking CCL20 to the recruitment of Th17 cells and the progression of EAP. During EAP development, CCL20 within the prostate can recruit Th17 cells, while IL‐17A, secreted by these cells, activates macrophages via the NF‐κB/MAPK/PI3K pathway to produce additional CCL20, thus establishing a positive feedback loop amplifying the inflammatory response. This research highlights the importance of CCL20 as a key mediator in prostatitis pathogenesis, contributing to inflammatory cell infiltration. CCL20 holds promise as a potential target for prostatitis treatment and a novel diagnostic marker. Nevertheless, extensive clinical studies are essential to validate the clinical utility of CCL20 in prostatitis management.

## AUTHOR CONTRIBUTIONS

**Cheng Zhang:** Conceptualization (equal); data curation (equal); formal analysis (equal); investigation (equal); methodology (equal); resources (equal); software (equal); visualization (equal); writing – original draft (equal). **Shun Xu:** Formal analysis (equal); investigation (equal); software (equal); validation (equal); visualization (equal). **Rui‐Jie Hu:** Investigation (equal); validation (equal); visualization (equal). **Xian‐Hong Liu:** Investigation (equal); validation (equal). **Shao‐Yu Yue:** Investigation (equal); validation (equal). **Xiao‐Ling Li:** Investigation (equal); validation (equal). **Bang‐Shun Dai:** Investigation (equal); validation (equal). **Chao‐Zhao Liang:** Conceptualization (equal); funding acquisition (equal). **Chang‐Sheng Zhan:** Conceptualization (equal); funding acquisition (equal); project administration (equal); resources (equal); writing – review and editing (equal).

## FUNDING INFORMATION

This work received support from the National Natural Science Foundation of China (NSFC) (82000720, 82370776), as well as from the Natural Science Foundation of Anhui Province (2008085QH360).

## CONFLICT OF INTEREST STATEMENT

The authors herein affirmatively attest to the absence of any conflicts of interest pertinent to the subject matter expounded within the confines of this scholarly manuscript.

## Supporting information


Figure S1.

Table S1.

## Data Availability

Some of the original data was shown in the article/Supplement Material. If needed, the remaining data can be obtained by contacting the corresponding author.
