# Correction to “Unraveling CCL20's Role by Regulating Th17 Cell Chemotaxis in Experimental Autoimmune Prostatitis”

**DOI:** 10.1111/jcmm.70243

**Published:** 2024-12-27

C. Zhang, S. Xu, R. J. Hu, et al., “Unraveling CCL20’s Role by Regulating Th17 Cell Chemotaxis in Experimental Autoimmune Prostatitis,” *Journal of Cellular and Molecular Medicine* 28, no. 10 (2024): e18445.

In Cheng Zhang et al., the images for immunohistochemical staining of IL‐17A in Figure 4B were used incorrectly due to technical error during image preparation. The correct figure is shown below. The authors confirm all results and conclusions of this article remain unchanged.

**FIGURE 4:**
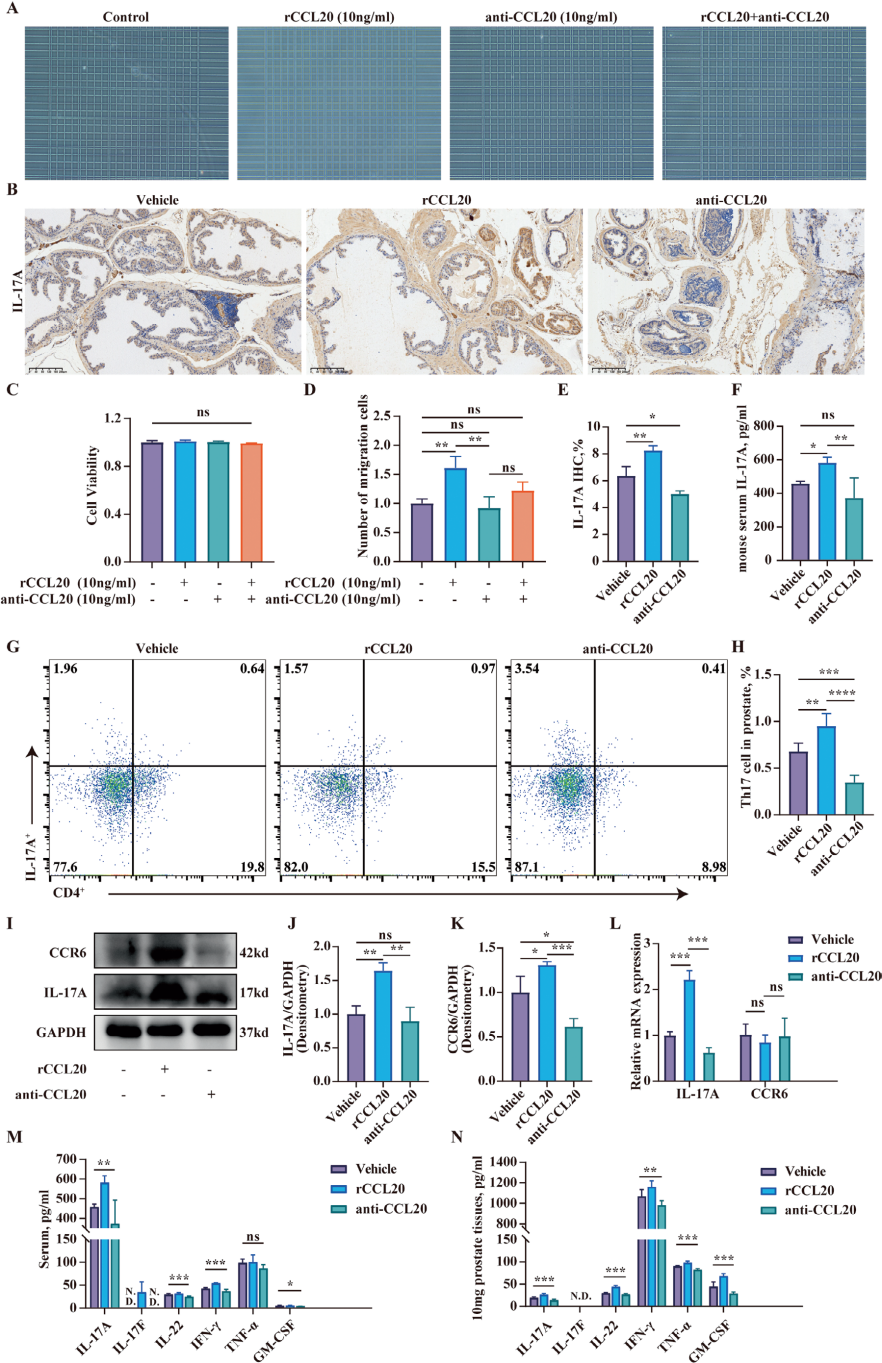
The recruitment of Th17 cells by CCL20. (A and D) Counting Th17 cells chemoattracted by CCL20 through transwell assay. (B) Immunohistochemical staining of IL‐17A on paraffin‐embedded prostate sections in each group. (C) Assessment of cell viability of Th17 cells after treatment with rCCL20 or anti‐CCL20 neutralising antibodies. (E) Semi‐quantitative analysis of CCL20 in immunohistochemical staining. (F) Quantitative analysis of IL‐17A in the serum of mice determined by ELISA. (G–H) Flow cytometry analysis of CD4^+^IL‐17A^+^ cells in the prostate. (I–K) The expression level of IL‐17A and CCR6 in the prostate tissues determined by western blot. (L) Relative expression of IL‐17A and CCR6 in the prostate measured by RT‐qPCR. Representative data from three independent experiments are shown. Data are presented as mean ± SD and were analysed using one‐way ANOVA analysis. ‘ns’ *p* > 0.05; *p < 0.05; **p < 0.01; ***p < 0.001.

