# Supplementary figures and images for: Unraveling CCL20's role by regulating Th17 cell chemotaxis in experimental autoimmune prostatitis
Source: J Cell Mol Med. 2024 May 27;28(10):e18445. doi: 10.1111/jcmm.18445 (PMC11129727; doi:10.1111/jcmm.18445)

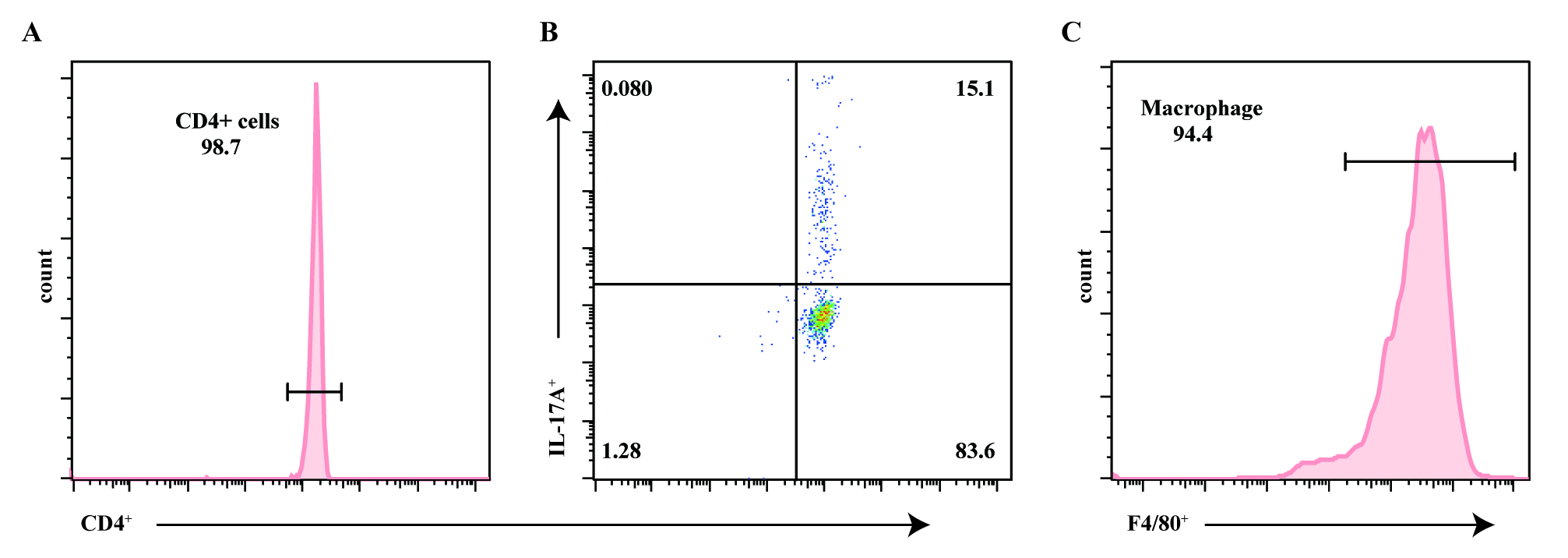

Supplement: Supplementary file 1 — Figure S1. [file JCMM-28-e18445-s002.tif]
