# Supplementary material for: Unraveling CCL20's role by regulating Th17 cell chemotaxis in experimental autoimmune prostatitis
Source: J Cell Mol Med. 2024 May 27;28(10):e18445. doi: 10.1111/jcmm.18445 (PMC11129727; doi:10.1111/jcmm.18445)
Supplement: Supplementary file 2 — Table S1. [file JCMM-28-e18445-s001.docx]

**Supplementary Table 1. Primers used for qPCR**

| **primer** | **Sequence (5’ to 3’)** |
| --- | --- |
| GAPDHF | GGTTGTCTCCTGCGACTTCA |
| GAPDHR | TGGTCCAGGGTTTCTTACTCC |
| CCL20F | TCTTCCTTGCTTTGGCATGGG |
| CCL20R | CAGTCGTAGTTGCTTGCTTCTG |
| CCR6F | GGTATGGGACTGGAGCTGTT |
| CCR6R | GATTGCTCTGTGCCTCTTGG |
| IL17AF | GCGATCATCCCTCAAAGCTC |
| IL17AR | TCTTCATTGCGGTGGAGAGT |
